# Supplementary material for: The causal role of circulating inflammatory markers in osteoporosis: a bidirectional Mendelian randomized study
Source: Front Immunol. 2024 Jul 18;15:1412298. doi: 10.3389/fimmu.2024.1412298 (PMC11291241; doi:10.3389/fimmu.2024.1412298)
Supplement: Supplementary file 1 [file DataSheet_1.zip › supplementary material/CIM-OSTEOPOROSIS /GCST90274841.csv_leave_one_out.pdf]

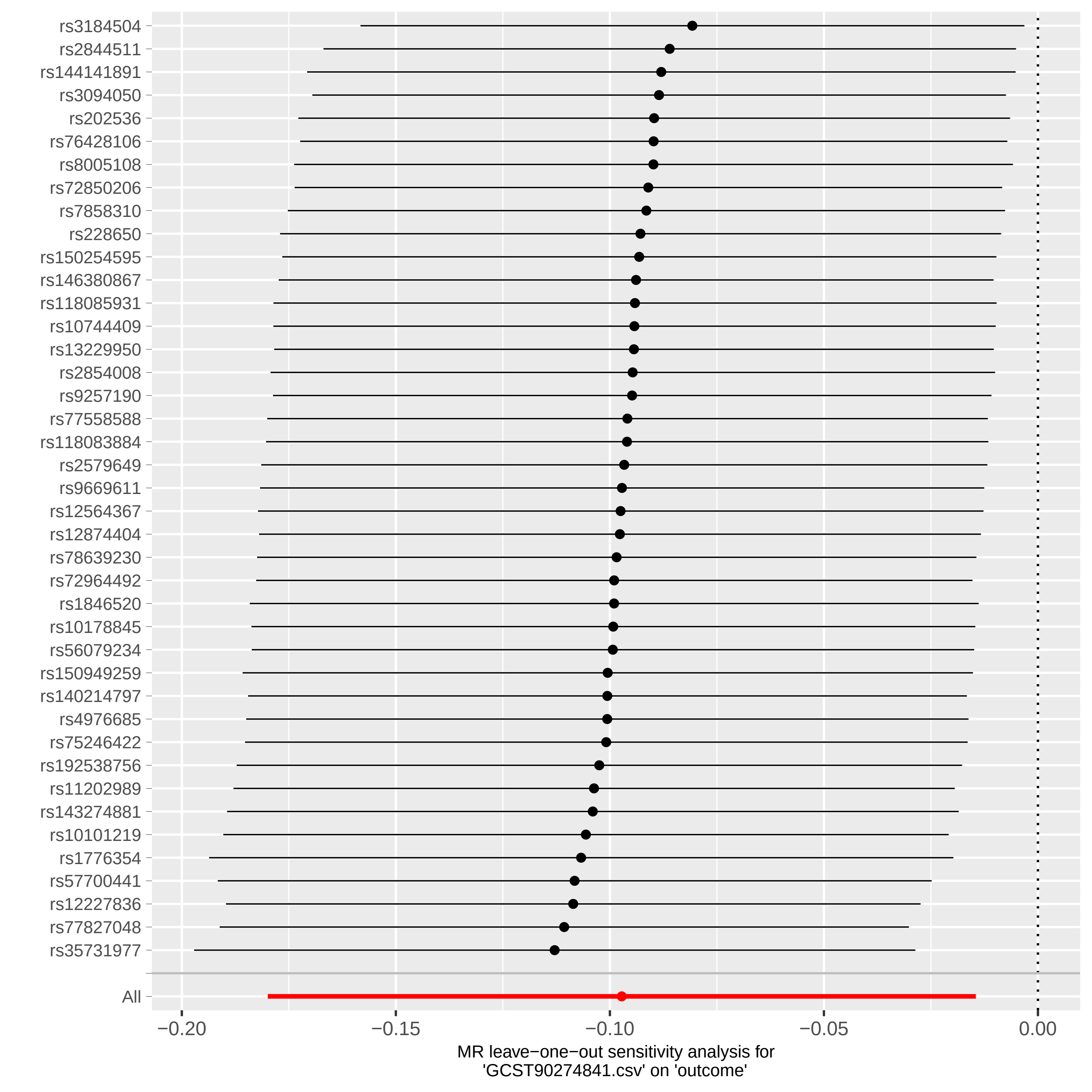
rs3184504
rs2844511
rs144141891
rs3094050
rs202536
rs76428106
rs8005108
rs72850206
rs7858310
rs228650
rs150254595
rs146380867
rs118085931
rs10744409
rs13229950
rs2854008
rs9257190
rs77558588
rs118083884
rs2579649
rs9669611
rs12564367
rs12874404
rs78639230
rs72964492
rs1846520
rs10178845
rs56079234
rs150949259
rs140214797
rs4976685
rs75246422
rs192538756
rs11202989
rs143274881
rs10101219
rs1776354
rs57700441
rs12227836
rs77827048
rs35731977
All
−0.20
−0.15
−0.10
−0.05
0.00
MR leave−one−out sensitivity analysis for
'GCST90274841.csv' on 'outcome'
